# Effects of Controlled-Release Fertilizer Application Rate on Growth, Physiological Traits, and Chlorophyll Fluorescence Responses of *Paeonia delavayi* Seedlings

**DOI:** 10.3390/plants15101525

**Published:** 2026-05-16

**Authors:** Haizhen Tong, Guiqing He, Shuang Li, Yunfei Huang, Yue Pan, Juan Wang

**Affiliations:** 1 College of Biological Science and Food Engineering, Southwest Forestry University, Kunming 650224, China; thz@swfu.edu.cn; 2 College of Landscape and Horticulture, Southwest Forestry University, Kunming 650224, China; heguiqing2013@126.com; 3 Institute of Alpine Economic Plants, Yunnan Academy of Agricultural Sciences, Lijiang 674100, China; 4 College of Forestry, Southwest Forestry University, Kunming 650224, China; aws0406@163.com (S.L.); 18248608637@163.com (Y.H.); panyue@163.com (Y.P.)

**Keywords:** biomass allocation, antioxidant enzymes, carbon assimilates, fuzzy membership analysis, container nursery

## Abstract

Controlled-release fertilizer (CRF) improves fertilizer-use efficiency through sustained nutrient release, but its rate-dependent effects on the growth and physiology of *Paeonia delavayi* seedlings remain unclear. In this study, germinated seeds of *P. delavayi* with radicles 3–4 cm in length were grown under container nursery conditions with four CRF application rates: control (CK, 0 kg·m^−3^), treatment 1 (T1, 0.6 kg·m^−3^), treatment 2 (T2, 1.2 kg·m^−3^), and treatment 3 (T3, 2.4 kg·m^−3^). Morphological traits, root characteristics, biomass accumulation, physiological parameters, and chlorophyll fluorescence were evaluated, and Pearson correlation and fuzzy membership analyses were used to compare overall treatment performance within the tested range. CRF significantly promoted seedling height, leaf number, petiole length, and biomass accumulation, although the promoting effect did not increase continuously with fertilizer rate. By June, seedling height in T2 was 160% greater than that in CK, while aboveground biomass increased by 552% and 574% in T2 and T3, respectively. Root morphological traits were not significantly affected, suggesting that CRF primarily promoted aboveground development and biomass production. Medium and high CRF rates increased leaf superoxide dismutase (SOD) activity by 42% and 103%, respectively, and peroxidase (POD) activity by 163% and 250%, respectively. Aboveground starch content was 45% higher in T2 than in CK. In contrast, photosynthetic pigment contents and the chlorophyll a/b ratio were not significantly affected by CRF. Chlorophyll fluorescence analysis showed that Fv/Fm remained stable among CRF treatments (0.78–0.82) and was significantly higher than that in CK (0.65), whereas the actual quantum yield of PSII [Y(II)] did not differ significantly among treatments. Relative to CK, the quantum yield of non-photochemical quenching [Y(NPQ)] increased from 0.20 to 0.40 in T2, while the quantum yield of non-regulated energy dissipation in PSII [Y(NO)] decreased from 0.37 to 0.24–0.22 in T2–T3. Pearson correlation and fuzzy membership analyses ranked the treatments as T2 > T3 > T1 > CK, indicating that T2 performed most favorably within the tested range, although its advantage over T3 was small. Overall, an appropriate CRF rate promoted *P. delavayi* seedling growth and was associated with changes in biomass accumulation, antioxidant enzyme activity, carbon assimilate storage, and chlorophyll fluorescence parameters.

## 1. Introduction

*Paeonia delavayi* belongs to the family Paeoniaceae, genus *Paeonia*, subgenus *Moutan* [1]. It is mainly distributed in open woodlands, shrublands, and grassy slopes at elevations of 1900–4000 m in southeastern Tibet, north-central Yunnan, and western Sichuan, China [2]. Owing to its remarkable variation in flower color, *P. delavayi* represents an important germplasm resource for breeding new tree peony cultivars [3]. In addition, different tissues of *P. delavayi* contain diverse bioactive compounds, including flavonoids, monoterpene glycosides, and triterpenoids, and have shown potential hypoglycemic activity, suggesting promising medicinal and functional applications [4]. However, with the decline of wild populations and increasing conservation demands, artificial propagation has become a key strategy for the protection and sustainable utilization of this species [5]. The seedling stage is critical for the formation of seedling quality and the establishment of subsequent growth potential. Inadequate or improper nutrient supply during this stage may suppress current growth, negatively affect later development, and reduce the ornamental and economic value of the plants [6,7]. Therefore, clarifying nutrient responses during the seedling stage and establishing an appropriate nutrient management strategy are essential for the cultivation of *P. delavayi*.

Nitrogen (N), phosphorus (P), and potassium (K) are the three primary mineral nutrients required for plant growth and development. N plays an important role in chlorophyll biosynthesis, protein metabolism, and the accumulation of photosynthetic assimilates. P is essential for energy transfer, root development, and nutrient uptake. K enhances plant stress tolerance by regulating enzyme activity, osmotic balance, and stomatal movement [8]. In nursery production, conventional basal fertilizers can improve the physicochemical properties of substrates or soils to some extent, but the capacity to supply readily available nutrients is often limited and may not adequately meet the changing nutrient demands of growing seedlings. As a result, additional fertilization is usually required according to plant developmental stages [9,10]. At the same time, conventional soluble N, P, and K fertilizers release nutrients rapidly after application, making them prone to leaching and volatilization. This reduces fertilizer-use efficiency and increases fertilization frequency and production costs [11].

Controlled-release fertilizer (CRF) can release nutrients steadily over an extended period and continuously meet plant demand for N, P, and K, offering advantages such as prolonged nutrient supply, improved nutrient-use efficiency, and reduced fertilization frequency [12]. Consequently, CRFs have attracted considerable attention in intensive cultivation systems and sustainable agriculture. Previous studies and reviews have shown that CRFs can improve fertilizer-use efficiency by delaying nutrient release, enhancing nutrient retention, and reducing leaching losses, thereby contributing to both yield improvement and environmental benefits across various cropping systems [13,14]. Beyond field crops, appropriate CRF application rates have also been shown to promote plant growth, biomass accumulation, and certain physiological traits, such as chlorophyll content, nutrient accumulation, and overall seedling quality [15,16]. However, the magnitude of plant responses and the optimal CRF application rates vary among species. Previous studies have demonstrated clear species differences in the positive effects of CRF on seedling height, leaf area, chlorophyll content, biomass, and nursery stock quality, with different woody and horticultural species exhibiting different optimal application rates [5]. Therefore, determining the appropriate CRF rate for specific species and nursery systems is essential for improving seedling quality and optimizing nutrient management.

To date, fertilizer studies in peony species have mainly focused on the combined application of soluble N, P, and K fertilizers and foliar application of macro-, meso-, and micronutrients. These studies have primarily examined their effects on plant growth, physiological traits, yield formation, and nutrient use, with oil peony ‘Fengdan’ (*Paeonia ostii* ‘Fengdan’) and several potted peony cultivars being the main study materials. Previous work has shown that appropriate combinations of N, P, and K can significantly increase the photosynthetic rate of ‘Fengdan’ and promote both seed yield and oil production [17,18]. In the potted peony cultivar ‘Zi Er Qiao’, suitable rates and ratios of N, P_2_O_5_, and K_2_O can coordinately enhance vegetative and reproductive growth and improve overall plant performance [19]. In addition to soil fertilization, foliar studies have shown that the timing of nitrogen spraying can affect biomass allocation and nutrient-use efficiency in ‘Fengdan’ shoots [20], whereas foliar application of boron, zinc, and other mineral nutrients can improve photosynthetic traits and nutrient accumulation [21]. Overall, current fertilizer studies in peony species have mainly focused on mature plants under field or pot cultivation, with fertilization practices primarily involving soluble fertilizers or foliar application. By contrast, studies on the effects of CRF during the seedling stage, particularly under container nursery conditions, remain limited in *Paeonia* species and are especially scarce for *P. delavayi*.

In this context, the present study evaluated the effects of different CRF application rates on the growth, physiological traits, and chlorophyll fluorescence responses of *P. delavayi* seedlings under container nursery conditions. This study aimed to clarify the response pattern of *P. delavayi* seedlings to different CRF levels, identify the optimal fertilization rate, and provide a scientific basis for nutrient management and high-quality container seedling production of this species.

## 2. Results

### 2.1. Effects of Different CRF Rates on Seedling Growth of Paeonia delavayi

#### 2.1.1. Morphological Growth Traits

**Figure 1:**
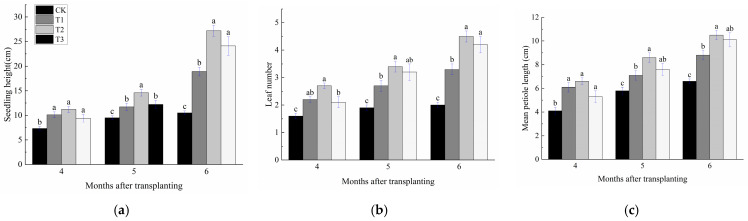
Effects of different controlled-release fertilizer rates (CRF) on seedling height (**a**), leaf number (**b**), and mean petiole length (**c**) of *Paeonia delavayi* seedlings at 4, 5, and 6 months after transplanting. Values are means ± SE (n = 12). Different lowercase letters indicate significant differences among treatments within the same month (*p* < 0.05).

Different CRF application rates significantly affected seedling height, leaf number, and mean petiole length in *P. delavayi* seedlings (Figure 1). All fertilized treatments improved these morphological growth traits relative to CK across the three measurement dates, with the most pronounced promoting effect observed under the T2 treatment (1.2 kg·m^−3^).

At 4 months after transplanting, seedling height in T1, T2, and T3 increased by 38.46%, 53.33%, and 29.87%, respectively, compared with CK. Leaf number increased by 44.52%, 76.13%, and 34.19%, respectively, and mean petiole length increased by 48.79%, 62.33%, and 33.58%, respectively. Among the fertilized treatments, no significant differences were observed in seedling height or mean petiole length, whereas leaf number in T2 was significantly higher than that in T3. At 5 months after transplanting, seedling height in T1, T2, and T3 increased by 23.26%, 53.83%, and 28.67%, respectively, compared with CK. Leaf number increased by 43.32%, 83.96%, and 69.52%, respectively, and mean petiole length increased by 22.86%, 48.46%, and 30.95%, respectively. No significant differences were detected among fertilized treatments for leaf number or mean petiole length, whereas seedling height in T2 was significantly greater than that in T3. At 6 months after transplanting, seedling height in T1, T2, and T3 increased by 80.95%, 159.66%, and 129.92%, respectively, relative to CK. Leaf number increased by 66.00%, 127.50%, and 108.50%, respectively, and mean petiole length increased by 33.33%, 58.20%, and 52.97%, respectively. Seedling height and leaf number were significantly higher in both T2 and T3 than in T1, and mean petiole length was also significantly higher in T2 than in T1.

#### 2.1.2. Root Growth

Different CRF application rates did not significantly affect root morphology in *P. delavayi* seedlings (Table 1).

#### 2.1.3. Biomass Accumulation

Different CRF application rates significantly affected root biomass, leaf biomass, shoot biomass, and root-to-shoot ratio, whereas no significant effect was observed on stem biomass (Table 2). Compared with CK, root biomass increased by 156.20%, 277.12%, and 252.50% under T1, T2, and T3, respectively. Leaf biomass increased by 287.53%, 561.73%, and 600.57%, respectively, while shoot biomass increased by 278.62%, 545.07%, and 567.78%, respectively. Stem biomass was significantly higher than that in CK only under T2, increasing by 392.28%.

Among the fertilized treatments, root biomass, leaf biomass, and shoot biomass were significantly greater in T2 and T3 than in T1, whereas no significant differences were observed between T2 and T3. Stem biomass did not differ significantly among fertilized treatments. In addition, root-to-shoot ratio decreased by 30.41%, 35.02%, and 47.00% under T1, T2, and T3, respectively, compared with CK, although no significant differences were observed among fertilized treatments.

### 2.2. Effects of Different CRF Rates on Physiological Traits of Paeonia delavayi Seedlings

#### 2.2.1. POD and SOD Activities

**Figure 2:**
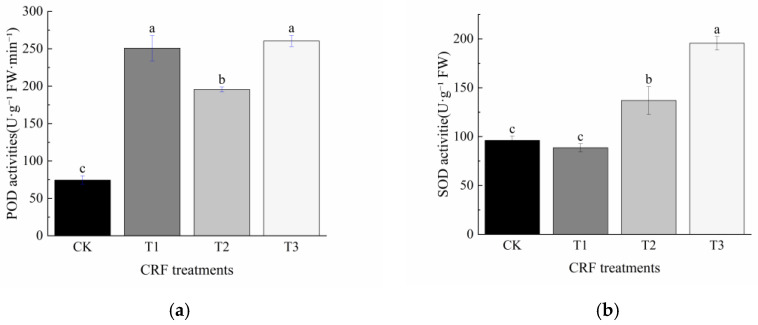
Effects of controlled-release fertilizer rate on (**a**) POD activity and (**b**) SOD activity in *P. delavayi* seedlings. Values are means ± SE (n = 12). Different lowercase letters indicate significant differences among treatments within the same month (*p* < 0.05).

Different CRF application rates significantly affected peroxidase (POD) and superoxide dismutase (SOD) activities in the leaves of *P. delavayi* seedlings (Figure 2). Compared with CK, POD activity increased by 237.10%, 162.90%, and 250.00% under T1, T2, and T3, respectively, although no significant differences were detected among the fertilized treatments. SOD activity in T1 did not differ significantly from that in CK, whereas it increased by 42.41% and 103.26% in T2 and T3, respectively. Among the fertilized treatments, SOD activity was significantly higher in T3 than in T2, and significantly higher in T2 than in T1.

#### 2.2.2. Non-Structural Carbohydrates and Soluble Protein Contents

The effects of CRF application rate on non-structural carbohydrate and soluble protein contents varied among indicators and plant organs (Table 3). CRF did not significantly affect soluble sugar or soluble protein contents in the aboveground parts, or starch content in the belowground parts. By contrast, it significantly affected starch content in the aboveground parts and soluble sugar content in the belowground parts. In addition, soluble protein content in the belowground parts under T1 was significantly lower than that in CK.

Compared with CK, starch content in the aboveground parts was significantly higher under T1, T2, and T3, with the highest value observed in T2, which represented an increase of 44.46%; however, no significant differences were detected among the fertilized treatments. Soluble sugar content in the belowground parts decreased by 15.60%, 44.42%, and 42.78% under T1, T2, and T3, respectively, compared with CK, and both T2 and T3 were significantly lower than those in T1.

#### 2.2.3. Photosynthetic Pigments

Different CRF application rates did not significantly affect chlorophyll a, chlorophyll b, total chlorophyll, chlorophyll a/b, or carotenoid contents in the leaves of *P. delavayi* seedlings (Table 4).

### 2.3. Effects of Different CRF Rates on Chlorophyll Fluorescence Characteristics of Paeonia delavayi Seedlings

The effects of different CRF application rates on chlorophyll fluorescence parameters differed among traits (Table 5). Among the measured parameters, the actual quantum yield of PSII [Y(II)] and the non-photochemical quenching coefficient (NPQ) did not differ significantly among treatments, whereas the other fluorescence parameters were significantly affected. Overall, however, differences among fertilized treatments were relatively small.

Compared with CK, CRF treatments significantly increased initial fluorescence (F_0_), maximum fluorescence (Fm), the maximum quantum yield of PSII (Fv/Fm), and the quantum yield of regulated energy dissipation in PSII [Y(NPQ)], with the highest values observed under T2. Under T2, F_0_, Fm, Fv/Fm, and Y(NPQ) increased by 67.59%, 120.55%, 26.15%, and 100.00%, respectively, compared with CK. The photochemical quenching coefficient (qP) was significantly lower under T1 and T2 than under CK, whereas no significant difference was detected between T3 and CK. The lowest qP value was observed under T2, representing a value 35.63% lower than that of CK. In addition, the quantum yield of non-regulated energy dissipation in PSII [Y(NO)] was significantly lower under all CRF treatments than under CK, with no significant differences among fertilized treatments. The lowest Y(NO) value occurred under T3, representing a 40.54% reduction compared with CK.

### 2.4. Correlation Analysis of Growth, Physiological, and Photosynthesis-Related Traits in Paeonia delavayi Seedlings

**Figure 3:**
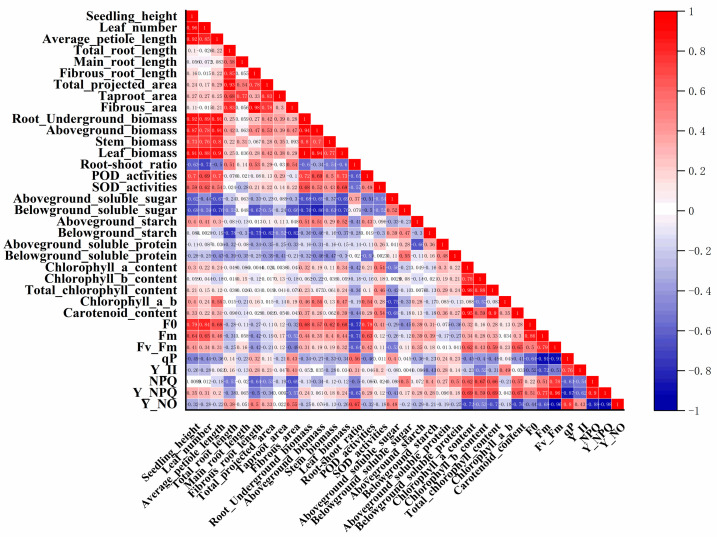
Pearson correlation analysis of the measured traits in *P. delavayi* seedlings. The color gradient of the triangular matrix represents the Pearson correlation coefficient, ranging from −1.0 (blue, strong negative correlation) to 1.0 (red, strong positive correlation). The numerical values in the matrix indicate the corresponding Pearson correlation coefficients.

To explore relationships among growth, physiological, and photosynthesis-related traits in *P. delavayi* seedlings, Pearson correlation analysis was performed (Figure 3). Overall, correlations of varying strengths were detected among the measured traits, and most growth-related variables showed significant or highly significant positive correlations.

Among morphological growth and biomass traits, seedling height, leaf number, and mean petiole length were all positively correlated with each other and were also positively correlated with shoot biomass, root biomass, and leaf biomass. Root morphology traits also showed strong positive correlations, with total root length, taproot length, lateral root length, and root surface area all positively correlated with one another and with root biomass. With respect to the relationships between growth traits and physiological or photosynthetic traits, some morphological variables showed associations with antioxidant enzyme activities and chlorophyll fluorescence parameters. Among the photosynthetic pigment traits, chlorophyll a, chlorophyll b, and total chlorophyll were highly significantly positively correlated with each other and were also positively correlated with carotenoid content. Chlorophyll fluorescence traits were also clearly interrelated: Fv/Fm was positively correlated with qP and Y(II) but negatively correlated with NPQ and Y(NO).

### 2.5. Comprehensive Evaluation of CRF Treatments Using Fuzzy Membership Analysis in Paeonia delavayi Seedlings

The comprehensive fuzzy membership evaluation showed that the overall membership values ranked in the order of T2 > T3 > T1 > CK, indicating that T2 exhibited the best overall performance among all treatments. Compared with T3 and T1, the comprehensive membership value of T2 was increased by 16.67% and 42.19%, respectively (Table 6).

## 3. Discussion

### 3.1. Effects of CRF Application Rates on the Growth of Paeonia delavayi Seedlings

Seedling height, leaf number, petiole length, and biomass are key indicators reflecting seedling performance and nursery stock quality. In this study, CRF application significantly promoted the growth of *P. delavayi* seedlings within the tested range, but the promotive effect did not increase continuously with increasing fertilizer rates. The medium CRF level (T2) showed relatively more favorable overall performance, although its differences from the high CRF level (T3) were small for some growth-related traits. Similar patterns have been reported in other plant species, where moderate or species-appropriate CRF rates yield superior growth and nutrient-use efficiency [22]. In early-ripening rapeseed, CRF applied at 1500 kg·hm^−2^ produced the highest seed yield and economic return, while increasing yield and reducing fertilizer input compared with the corresponding soluble fertilizer treatment [23]. In a container nursery study of six woody ornamental shrubs, the acceptable CRF application range differed clearly among species, highlighting interspecific variation in fertilizer demand [5]. This pattern may be related to the nutrient-release characteristics of CRF and the efficiency with which plants utilize the released nutrients. By supplying nutrients gradually over an extended period, CRF can help maintain favorable growth and physiological performance under moderate nutrient input [24].

At the later sampling dates, the growth-promoting effect of CRF on *P. delavayi* seedlings became more pronounced at later growth stages. Compared with the control, the increases in seedling height and leaf number observed in June were markedly greater than those recorded earlier, with T2 showing a relatively stronger promotive effect. This pattern may be related to sustained nutrient-release characteristics of CRF, which could have better matched seedling nutrient demand during the later growth period. This agrees with previous reports showing that CRF can sustain favorable crop growth, improve nutrient-use efficiency, and better synchronize nutrient release with plant nutrient demand [15,24,25].

Biomass partitioning followed the order of leaf > root > stem, and significant differences among fertilization treatments were observed mainly in aboveground biomass, especially leaf biomass, with stronger promotion under medium and high CRF levels. A similar response to slow-release fertilizer has also been reported in *Pistacia chinensis* Bunge [26]. Because leaves are the primary photosynthetic organs, they are generally more responsive to nutrient availability. The increase in leaf biomass therefore suggests an expansion of photosynthetic area and a greater capacity for assimilate production, which would provide a stronger material basis for whole-plant growth.

Changes in the root-to-shoot ratio further reflected biomass allocation strategies under different nutrient conditions. Under the control treatment, limited nutrient availability in the substrate likely promoted a higher root-to-shoot ratio, thereby enhancing nutrient acquisition. In contrast, fertilization alleviated nutrient limitation and promoted greater allocation to aboveground organs, which favored leaf expansion and overall growth. This pattern is consistent with the general allocation strategy of plants under nutrient-deficient conditions [27]. Notably, CRF application had no significant effect on root morphological traits in this study. The root traits measured in this study focus only on macroscopic morphology. Assessed at merely one sampling stage, they may fail to capture subtle and dynamic root responses to CRF treatments. Furthermore, the fine roots of *P. delavayi* are extremely slender and fragile, prone to breakage and loss during root cleaning. Even with careful handling, such damage may have masked subtle differences among treatments. Beyond methodological constraints, this trend may also be associated with species-specific biological characteristics. Fertilization can significantly promote root growth in grass–legume mixed systems on the Qinghai–Tibet Plateau [28], while as a woody species, *P. delavayi* inherently shows a limited morphological root response to controlled-release fertilization.

As an ornamental and medicinal woody species, *P. delavayi* exhibits enhanced seedling height, leaf quantity and aboveground biomass under proper CRF supply. Such changes improve seedling quality and benefit subsequent transplant survival and cultivation performance.

### 3.2. Effects of CRF Application Rates on the Physiological Traits of Paeonia delavayi Seedlings

Plants maintain intracellular reactive oxygen species (ROS) homeostasis through coordinated antioxidant systems, thereby reducing the risk of oxidative damage under environmental or nutritional stress [29,30]. Among these systems, SOD converts superoxide radicals (O_2_^−^) into H_2_O_2_, which is subsequently scavenged by POD and other antioxidant enzymes [30,31]. In this study, leaf SOD and POD activities were higher under medium and high CRF treatments, suggesting that CRF application was associated with higher antioxidant enzyme activity and may therefore have contributed to a relatively more stable physiological state in *P. delavayi* seedlings.

Soluble sugars and soluble proteins are important osmotic adjustment substances in plants [32]. In this study, the contents of soluble sugar and soluble protein in roots were significantly higher in the control treatment than in all fertilized treatments, suggesting that seedlings in the unfertilized treatment may have experienced stronger nutrient stress. Under such conditions, the accumulation of osmotic adjustment substances may help maintain osmotic balance and alleviate stress effects. After CRF application, nutrient supply became more adequate, and the demand for such stress-related osmotic adjustment appeared to decrease accordingly.

In addition, CRF significantly promoted starch accumulation in the aboveground parts of *P. delavayi* seedlings, and starch content under medium and high CRF levels (T2 and T3) was significantly higher than that under low CRF level (T1). This suggests that an appropriate CRF rate can facilitate the accumulation and conversion of photosynthetic products. A plausible explanation is that the sustained supply of N and P from CRF improved the functional status of photosynthetic organs and supported carbon assimilation. Meanwhile, CRF also increased leaf number and leaf biomass, which may have further enhanced total assimilate production [33,34]. However, no significant differences were detected among CRF treatments in photosynthetic pigment contents or chlorophyll a/b ratio, indicating that the physiological effects of CRF on *P. delavayi* seedlings were not primarily mediated through changes in photosynthetic pigment contents.

### 3.3. Effects of CRF Application Rates on Chlorophyll Fluorescence Characteristics in Paeonia delavayi Seedlings

Chlorophyll fluorescence parameters are important indicators of plant photosynthetic performance and energy dissipation [34,35]. In this study, leaf F_0_ was significantly higher in all CRF treatments than in CK, whereas no significant differences were detected among CRF treatments. Fm was significantly higher under T1 and T2 than under CK, whereas T3 showed an intermediate level and did not differ significantly from CK or the other fertilized treatments. Fv/Fm remained generally stable among the CRF treatments but was significantly higher than that in CK. This suggests that CRF application did not markedly alter the maximum potential photochemical efficiency of PSII among the fertilized treatments [36,37]. Given that chlorophyll content also remained unchanged, the increase in F_0_ is unlikely to indicate structural damage to PSII reaction centers but may instead reflect nutrient-related adjustments in excitation energy distribution. Overall, these results suggest that PSII status remained relatively stable among the CRF treatments, whereas the lower Fv/Fm value in CK indicates that the unfertilized seedlings may have experienced stronger stress under the present experimental conditions [38].

qP and Y(II) together reflect the actual efficiency of light utilization in PSII [38,39]. In this study, qP was significantly lower under T1 and T2 than under CK, whereas Y(II) did not differ significantly among treatments. This indicates that the reduction in qP was not accompanied by a decline in actual photochemical efficiency. Therefore, the change in qP was unlikely to reflect impairment of PSII function but may instead indicate an adjustment in the proportion of open reaction centers under improved nutrient supply. Together with the stable responses of F_0_, Fm, Fv/Fm, and Y(II), these findings suggest that the actual photochemical performance of PSII remained relatively stable among the CRF treatments.

NPQ and its quantum yield components [Y(NPQ) and Y(NO)] reflect the capacity of plants to dissipate excess excitation energy as heat and can be used to characterize PSII energy partitioning [39,40]. In the present study, NPQ did not differ significantly among treatments, indicating that CRF application did not markedly alter the total intensity of thermal dissipation. However, all CRF treatments significantly increased Y(NPQ) and significantly decreased Y(NO) relative to CK, while Y(II) remained stable. These results suggest that CRF application did not increase the overall level of thermal dissipation but was associated with a shift in the energy dissipation pattern, with a greater proportion of regulated energy dissipation and a lower proportion of passive energy loss. This pattern suggests that CRF application may have improved photoprotective regulation in PSII in *P. delavayi* seedlings.

It should be noted that the relatively higher variability observed in CK seedlings may be associated with differences in leaf developmental stage and the limited number of leaves available for measurement. These factors could have introduced additional variation in chlorophyll fluorescence parameters under CK conditions.

### 3.4. Correlation and Fuzzy Membership Function Analyses

Pearson correlation analysis revealed different degrees of association among trait categories in *P. delavayi* seedlings, with the strongest correlations occurring between morphological growth traits and biomass accumulation. Seedling height, leaf number, and average petiole length were significantly or highly significantly positively correlated with one another and were also strongly positively correlated with shoot biomass, leaf biomass, and belowground biomass. By contrast, the root-to-shoot ratio was generally negatively correlated with seedling height, leaf number, and shoot biomass, indicating that improved nutrient supply shifted biomass allocation toward aboveground growth. In addition, aboveground starch content was generally positively correlated with several growth traits, whereas belowground soluble sugar was generally negatively correlated with some growth traits, suggesting that CRF-promoted growth was accompanied by increased assimilate accumulation and reduced accumulation of stress-related substances in roots.

Photosynthetic pigment traits were strongly positively correlated with one another, whereas their correlations with growth traits were comparatively weak, which is consistent with the absence of significant differences in pigment contents among fertilization treatments. Chlorophyll fluorescence parameters also showed clear interrelationships. Fv/Fm was positively correlated with qP and Y(II), but negatively correlated with Y(NO), whereas Y(NPQ) was negatively correlated with Y(NO), indicating close coordination between PSII performance and energy dissipation pattern. Combined with the fluorescence results described above, these relationships suggest that the potential and actual photochemical efficiencies of PSII remained largely stable under CRF treatment, whereas the differences among treatments were mainly reflected in the partitioning pattern of excess excitation energy.

Fuzzy membership function analysis further showed that the overall performance ranked as T2 > T3 > T1 > CK. This ranking was generally consistent with the responses of the individual growth, physiological, and fluorescence traits, indicating that T2 showed relatively more favorable overall performance under the selected indicator system and the present experimental conditions.

It should be noted that this study was conducted under specific greenhouse, substrate, and container nursery conditions, and therefore the results mainly reflect the responses of *P. delavayi* seedlings to CRF under the present experimental conditions. Differences in substrate composition, fertilizer formulation, container volume, and cultivation season may all influence the appropriate application level. In addition, the CRF rate gradients used in this study were primarily intended to compare the relative differences among treatments, and thus the present results are more suitable for evaluating the overall performance of the treatments within the tested range. Meanwhile, the measurements of root morphology and some physiological traits were mainly based on a single sampling stage and may not fully reflect their dynamic variation. Greenhouse environmental factors were not continuously monitored during the experiment, and temporal fluctuations in environmental conditions, such as light intensity and temperature, may have influenced seedling physiological traits and chlorophyll fluorescence parameters. These fluctuations may affect photosynthetic performance by altering light energy absorption and utilization efficiency, as well as by modulating enzymatic activities and energy dissipation in the photosynthetic apparatus [41,42]. However, since all treatments were conducted simultaneously under the same open greenhouse conditions, such environmental variations are expected to have exerted comparable effects across treatments. Therefore, these fluctuations may be considered largely non-systematic and are unlikely to bias the relative differences among treatments.

## 4. Materials and Methods

### 4.1. Experimental Site and Plant Materials

The experiment was conducted in a greenhouse at the Arboretum of Southwest Forestry University, Kunming, Yunnan Province, China (25°03′ N, 102°46′ E). The region has a subtropical plateau monsoon climate, with a mean annual temperature of approximately 14.7 °C and mean annual precipitation of approximately 943 mm. Rainfall is mainly concentrated from May to September, whereas the dry season extends from November to April and accounts for only about 15% of the annual precipitation. The frost-free period exceeds 240 days, and the annual sunshine duration averages 2445.6 h, with a sunshine percentage of approximately 56%.

Seeds of *P. delavayi* were used as the plant material. On 1 October 2021, the seeds were subjected to moist sand stratification for germination in the greenhouse. On 23 December 2021, germinated seeds with radicles 3–4 cm in length were selected and soaked in 300 mg·L^−1^ gibberellic acid (GA_3_, Coolaber Technology Co., Ltd., Beijing, China) solution for 45 min before transplanting. The nursery substrate consisted of red soil, peat, and perlite mixed at a volume ratio of 3:1:1. Its physicochemical properties were as follows: pH 6.40, electrical conductivity 1.11 μS·cm^−1^, bulk density 0.82 g·cm^−3^, organic matter 41.64 g·kg^−1^, total nitrogen 1.22 g·kg^−1^, total phosphorus 0.49 g·kg^−1^, and total potassium 5.92 g·kg^−1^. Before use, the substrate was disinfected with 0.5% potassium permanganate solution for 48 h. The fertilizer used in this study was a compound controlled-release fertilizer (Haifa Chemicals Ltd., Haifa, Israel) containing 18.4% N, 6.3% P_2_O_5_, and 12.3% K_2_O, with a nutrient release period of 6 months.

### 4.2. Experimental Design

A single-factor randomized block design was adopted, with four CRF application rates incorporated into the nursery substrate: CK (0 kg·m^−3^), T1 (0.6 kg·m^−3^), T2 (1.2 kg·m^−3^), and T3 (2.4 kg·m^−3^). Each treatment consisted of three replicates with 24 seedlings each (72 seedlings per treatment; 288 seedlings in total).

The CRF was thoroughly mixed with the substrate at the designated rates and then filled into nonwoven nursery bags. After filling, the bags were approximately 10.5 cm in diameter and 14 cm in height, with a substrate mass of 650 ± 20 g per bag. One germinated seed was transplanted into each bag, with care taken to keep the radicle intact, and the covering soil thickness was maintained at 1.0–1.5 cm. After transplanting, seedlings from different treatments were arranged uniformly in the greenhouse. Buffer bags filled with the same substrate were placed around the edge of the experiment to minimize border effects. All seedlings were then irrigated thoroughly and managed using standard nursery practices.

### 4.3. Measurements and Methods

#### 4.3.1. Growth Traits

Growth traits were measured three times on 23 April, 21 May, and 23 June 2022. Seedling height was measured with a steel tape from the substrate surface to the shoot apex. The petiole length of the first leaf above the stem base was also measured, and the number of leaves was recorded. For growth measurements, four seedlings were randomly selected from each replicate at each sampling date, giving 12 seedlings per treatment in total at each measurement.

#### 4.3.2. Root Morphology and Biomass

After the final growth measurements on 24 June 2022, seedlings were carefully removed from the bags and the roots were washed gently with tap water. After surface moisture was blotted off, each plant was separated into roots, stems, and leaves, and the fresh weight of each component was recorded. Root length and root surface area were measured using a WinRHIZO root analysis system. After measurement, each organ was placed in a labeled paper bag, heated at 110 °C for 45 min to deactivate enzymes, and then dried at 80 °C to constant weight, defined as a difference of no more than 0.001 g over three consecutive weighings. Root, stem, leaf, and total biomass were then calculated, together with the root-to-shoot ratio (root biomass/shoot biomass). For root morphology and biomass determination, another four seedlings were randomly selected from each replicate, giving 12 seedlings per treatment in total.

#### 4.3.3. Physiological Traits

In June 2022, 12 seedlings with uniform growth were selected from each treatment. The first leaf above the stem base was sampled, and every three seedlings were pooled as one biological replicate, resulting in four biological replicates per treatment. Samples were immediately frozen in liquid nitrogen after collection and stored at −80 °C for subsequent determination of antioxidant enzyme activities, non-structural carbon, and soluble protein contents. For physiological and photosynthetic pigment analyses, 12 seedlings were selected from each treatment. Every three seedlings were pooled as one biological replicate, resulting in four biological replicates per treatment.

All physiological parameters were determined according to the method described by Li [43]. Superoxide dismutase (SOD) activity was determined using the nitro blue tetrazolium (NBT) method, and peroxidase (POD) activity was measured using the guaiacol method. Soluble sugar and starch contents were determined by the anthrone colorimetric method, and soluble protein content was measured using the Coomassie Brilliant Blue G-250 method. Photosynthetic pigments were extracted with ethanol–acetone (1:1, *v*/*v*) and quantified spectrophotometrically, and the chlorophyll a/b ratio was subsequently calculated.

#### 4.3.4. Chlorophyll Fluorescence Parameters

On 24 June 2022, six seedlings were randomly selected from each treatment, and the first leaf above the stem base was used for chlorophyll fluorescence measurements. A portable chlorophyll fluorometer (MINI-PAM-II, Walz, Germany) was used following the method of reference [44]. Leaves were dark-adapted for 30 min before measurement. Photosynthetically active radiation (PAR) was applied in a stepwise gradient of 0, 35, 60, 94, 159, 253, 388, 594, 786, 1112, and 1458 μmol·m^−2^·s^−1^, with each light level maintained for 30 s. Steady-state fluorescence (Fs), minimum fluorescence in the dark-adapted state (F_0_), maximum fluorescence in the dark-adapted state (Fm), and minimum (F_0_′) and maximum (Fm′) fluorescence in the light-adapted state were recorded. Based on these measurements, the maximum quantum yield of PSII [Fv/Fm = (Fm − F_0_)/Fm], the effective quantum yield of PSII [Fv′/Fm′ = (Fm′ − F_0_′)/Fm′], the photochemical quenching coefficient [qP = (Fm′ − Fs)/(Fm′ − F_0_′)], and the non-photochemical quenching coefficient [NPQ = (Fm − Fm′)/Fm′] were calculated. The parameters Y(II), Y(NPQ), and Y(NO) were obtained directly from the instrument software. For chlorophyll fluorescence measurements, two seedlings were randomly selected from each replicate, giving six seedlings per treatment in total. Each seedling was treated as one biological replicate. One fully expanded healthy leaf was then selected from each seedling for measurement. When more than one fully expanded leaf was available, the uppermost leaf was selected. In CK seedlings, only one leaf was available due to limited leaf development and was directly used for measurement.

### 4.4. Data Processing and Statistical Analysis

Raw data were organized using WPS Office, statistical analyses were performed with SPSS 26.0, and figures were generated using Origin 2023. For growth traits, root morphology, biomass, and chlorophyll fluorescence parameters, individual seedlings served as the experimental unit in the statistical analysis. For physiological and photosynthetic pigment traits, three seedlings were pooled to form one biological replicate, and each pooled sample served as the experimental unit. Because growth measurements at different time points were based on independent seedling samples, growth traits were analyzed separately at each sampling date using one-way analysis of variance (one-way ANOVA). Other measured variables were also analyzed by one-way ANOVA. Before ANOVA, data were tested for normality and homogeneity of variance to ensure that the assumptions of the analysis were satisfied. When treatment effects were significant, Duncan’s multiple range test was used for mean comparisons at *p* < 0.05. Comprehensive evaluation was conducted using the fuzzy membership function method. Only variables that showed significant responses to fertilization treatment (*p* < 0.05) and had a clear positive or negative interpretive direction were included in the analysis. These selected variables were considered to represent treatment-related differences in seedling growth, biomass accumulation, physiological response, and chlorophyll fluorescence. Variables without significant treatment effects or without clear directional meaning for performance evaluation were excluded to improve the interpretability of the comprehensive ranking. For positive indicators: μij = (X_ij_ − X_min_)/(X_max_ − X_min_); For negative indicators: μij = 1 − μ_ij_ = (X_ij_ − X_min_)/(X_max_ − X_min_). where μ_ij_ is the membership value of the jth indicator under the *ith* treatment, X_ij_ is the measured value of that indicator under the corresponding treatment, and X_max_ and X_min_ are the maximum and minimum values of that indicator across all treatments, respectively.

## 5. Conclusions

The present study showed that CRF application significantly promoted the growth of *P. delavayi* seedlings and was associated with changes in physiological traits and light-energy utilization. Under the present experimental conditions, the moderate rate of 1.2 kg·m^−3^ (T2) showed relatively more favorable overall performance in terms of growth, biomass accumulation, physiological traits, and photosynthetic stability within the tested range. CRF application did not significantly affect the maximum photochemical efficiency of PSII but was associated with a shift in excitation energy partitioning by increasing regulated energy dissipation Y(NPQ) and decreasing non-regulated dissipation Y(NO). Overall, 1.2 kg·m^−3^ can be considered an appropriate CRF application rate for *P. delavayi* container seedlings under the present experimental conditions, providing a reference for nutrient management and high-quality seedling cultivation.

## Figures and Tables

**Table 1:** Root morphology of *P. delavayi* seedlings under different controlled-release fertilizer rates.

Treatment	Total Root Length mm	Main Root Length mm	Fibrous Root Length mm	Taproot Area cm^2^	Fibrous Area cm^2^
CK	393.57 ± 16.87 a	76.91 ± 10.26 a	316.66 ± 12.48 a	9.35 ± 0.86 a	20.23 ± 0.56 a
T1	362.33 ± 24.18 a	75.22 ± 10.26 a	287.13 ± 17.88 a	9.64 ± 1.02 a	18.92 ± 0.56 a
T2	407.80 ± 17.28 a	77.40 ± 14.14 a	330.41 ± 9.38 a	10.55 ± 0.80 a	20.44 ± 0.31 a
T3	379.21 ± 25.48 a	63.58 ± 9.44 a	315.63 ± 20.33 a	10.20 ± 0.86 a	20.23 ± 0.76 a

Values are means ± SE (n = 12). Different lowercase letters within the same column indicate significant differences among treatments at *p* < 0.05.

**Table 2:** Biomass accumulation and allocation in *P. delavayi* seedlings under different controlled-release fertilizer rates.

Treatment	Root (Belowground) Biomass g	Stem Biomass g	Leaf Biomass g	Aboveground Biomass g	Root-Shoot Ratio
CK	0.67 ± 0.11 c	0.03 ± 0.00 b	0.28 ± 0.03 c	0.31 ± 0.04 c	2.17 ± 0.25 a
T1	1.72 ± 0.29 b	0.09 ± 0.02 ab	1.09 ± 0.19 b	1.18 ± 0.20 b	1.51 ± 0.16 b
T2	2.53 ± 0.31 a	0.15 ± 0.05 a	1.86 ± 0.30 a	2.02 ± 0.34 a	1.41 ± 0.05 b
T3	2.37 ± 0.46 a	0.11 ± 0.04 ab	1.97 ± 0.29 a	2.09 ± 0.32 a	1.15 ± 0.16 b

Values are means ± SE (n = 12). Different lowercase letters within the same column indicate significant differences among treatments at *p* < 0.05.

**Table 3:** Non-structural carbohydrate and soluble protein contents of *P. delavayi* seedlings under different CRF treatments.

Non-Structural Carbon Compounds and Soluble Protein Contents	Treatment	Aboveground mg·g^−1^	Belowground mg·g^−1^
soluble sugar	CK	33.67 ± 3.93 a	47.57 ± 6.84 a
	T1	31.34 ± 4.39 a	40.15 ± 5.99 b
	T2	25.44 ± 1.66 a	26.44 ± 1.18 c
	T3	23.81 ± 1.60 a	27.22 ± 3.93 c
starch	CK	31.08 ± 2.57 b	55.80 ± 3.64 a
	T1	41.39 ± 8.40 a	59.17 ± 10.46 a
	T2	44.90 ± 4.38 a	54.77 ± 3.09 a
	T3	40.38 ± 9.18 a	54.27 ± 5.30 a
soluble protein	CK	0.12 ± 0.02 a	0.13 ± 0.01 a
	T1	0.09 ± 0.01 a	0.09 ± 0.01 b
	T2	0.09 ± 0.01 a	0.11 ± 0.00 ab
	T3	0.12 ± 0.02 a	0.12 ± 0.01 a

Values are means ± SE (n = 12). Different lowercase letters within the same column indicate significant differences among treatments at *p* < 0.05.

**Table 4:** Photosynthetic pigment contents in *P. delavayi* seedlings under different CRF treatments.

Treatment	Chlorophyll a mg·g^−1^	Chlorophyll b mg·g^−1^	Total Chlorophyll mg·g^−1^	Chlorophyll a/b	Carotenoid mg·g^−1^
CK	1.03 ± 0.24 a	0.31 ± 0.10 a	1.34 ± 0.33 a	3.5 ± 0.38 a	0.26 ± 0.05 a
T1	0.99 ± 0.06 a	0.25 ± 0.04 a	1.24 ± 0.09 a	4.08 ± 0.45 a	0.26 ± 0.01 a
T2	1.13 ± 0.06 a	0.30 ± 0.02 a	1.43 ± 0.08 a	3.77 ± 0.18 a	0.28 ± 0.02 a
T3	1.29 ± 0.05 a	0.30 ± 0.01 a	1.59 ± 0.05 a	4.32 ± 0.21 a	0.34 ± 0.02 a

Values are means ± SE (n = 12). Different lowercase letters within the same column indicate significant differences among treatments at *p* < 0.05.

**Table 5:** Chlorophyll fluorescence parameters of *P. delavayi* seedlings under different CRF treatments.

Treatment	F0	Fm	Fv/Fm	qP	Y(II)	NPQ	Y(NPQ)	Y(NO)
CK	181.00 ± 12.53 b	783.33 ± 279.02 b	0.65 ± 0.17 b	0.87 ± 0.16 a	0.43 ± 0.03 a	1.55 ± 0.94 a	0.20 ± 0.23 b	0.37 ± 0.20 a
T1	300.67 ± 26.14 a	1721.00 ± 200.73 a	0.82 ± 0.01 a	0.59 ± 0.02 b	0.39 ± 0.02 a	1.54 ± 0.46 a	0.36 ± 0.06 a	0.25 ± 0.03 b
T2	303.33 ± 2.33 a	1727.67 ± 24.84 a	0.82 ± 0.00 a	0.56 ± 0.02 b	0.36 ± 0.02 a	1.63 ± 0.11 a	0.40 ± 0.02 a	0.24 ± 0.00 b
T3	296.00 ± 12.22 a	1373.33 ± 70.48 a	0.78 ± 0.01 a	0.75 ± 0.02 ab	0.44 ± 0.01 a	1.51 ± 0.03 a	0.34 ± 0.01 a	0.22 ± 0.00 b

Values are means ± SE (n = 6). Different lowercase letters within the same column indicate significant differences among treatments at *p* < 0.05.

**Table 6:** Results of fuzzy membership function evaluation under different controlled-release fertilizer treatments.

Treatment	CK	T1	T2	T3
Plant height	0.00	0.51	1.00	0.81
Number of leaves	0.00	0.52	1.00	0.85
Average petiole length	0.00	0.57	1.00	0.91
Plant biomass	0.00	0.54	1.00	0.97
POD activity	0.00	0.95	0.65	1.00
SOD activity	0.07	0.00	0.45	1.00
Aboveground starch content	0.00	0.75	1.00	0.67
F_m_	0.00	0.99	1.00	0.62
F_v_/F_m_	0.00	1.00	1.00	0.76
NPQ	0.29	0.23	1.00	0.00
Y(NPQ)	0.00	0.82	1.00	0.71
Y(NO)	0.00	0.80	0.87	1.00
Membership value	0.03	0.64	0.91	0.78
Rank	4	3	1	2

Membership value represents the comprehensive evaluation value calculated by the fuzzy membership function method, and all membership values are presented to two decimal places. Rank indicates the overall order of treatment performance, with 1 representing the best performance.

## Data Availability

The original contributions presented in this study are included in the article. Further inquiries can be directed to the corresponding author.
